# Natural Substances vs. Approved Drugs in the Treatment of Main Cardiovascular Disorders—Is There a Breakthrough?

**DOI:** 10.3390/antiox12122088

**Published:** 2023-12-07

**Authors:** Jelica Grujić-Milanović, Jovana Rajković, Sladjan Milanović, Vesna Jaćević, Zoran Miloradović, Lana Nežić, Radmila Novaković

**Affiliations:** 1 Institute for Medical Research, National Institute of the Republic of Serbia, Department of Cardiovascular Research, University of Belgrade, 11 000 Belgrade, Serbia; zokim@imi.bg.ac.rs; 2 Institute for Pharmacology, Clinical Pharmacology and Toxicology, Faculty of Medicine, University of Belgrade, 11 000 Belgrade, Serbia; 3 Institute for Medical Research, National Institute of the Republic of Serbia, Department for Biomechanics, Biomedical Engineering and Physics of Complex Systems, University of Belgrade, 11 000 Belgrade, Serbia; sladjan.milanovic@imi.bg.ac.rs; 4 Department for Experimental Toxicology and Pharmacology, National Poison Control Centre, Military Medical Academy, 11 000 Belgrade, Serbia; v_jacevic@yahoo.com; 5 Medical Faculty of the Military Medical Academy, University of Defense, 11 000 Belgrade, Serbia; 6 Department of Chemistry, Faculty of Science, University of Hradec Kralove, 500 002 Hradec Kralove, Czech Republic; 7 Department of Pharmacology, Toxicology and Clinical Pharmacology, Faculty of Medicine, University of Banja Luka, Save Mrkalja 14, 78000 Banja Luka, Bosnia and Herzegovina; lana.nezic@med.unibl.org; 8 Institute of Molecular Genetics and Genetic Engineering, Center for Genome Sequencing and Bioinformatics, University of Belgrade, 11 000 Belgrade, Serbia; radmila.novakovic@imgge.bg.ac

**Keywords:** cardiovascular diseases, oxidative stress, natural products, antioxidants

## Abstract

Cardiovascular diseases (CVDs) are a group of diseases with a very high rate of morbidity and mortality. The clinical presentation of CVDs can vary from asymptomatic to classic symptoms such as chest pain in patients with myocardial infarction. Current therapeutics for CVDs mainly target disease symptoms. The most common CVDs are coronary artery disease, acute myocardial infarction, atrial fibrillation, chronic heart failure, arterial hypertension, and valvular heart disease. In their treatment, conventional therapies and pharmacological therapies are used. However, the use of herbal medicines in the therapy of these diseases has also been reported in the literature, resulting in a need for critical evaluation of advances related to their use. Therefore, we carried out a narrative review of pharmacological and herbal therapeutic effects reported for these diseases. Data for this comprehensive review were obtained from electronic databases such as MedLine, PubMed, Web of Science, Scopus, and Google Scholar. Conventional therapy requires an individual approach to the patients, as when patients do not respond well, this often causes allergic effects or various other unwanted effects. Nowadays, medicinal plants as therapeutics are frequently used in different parts of the world. Preclinical/clinical pharmacology studies have confirmed that some bioactive compounds may have beneficial therapeutic effects in some common CVDs. The natural products analyzed in this review are promising phytochemicals for adjuvant and complementary drug candidates in CVDs pharmacotherapy, and some of them have already been approved by the FDA. There are insufficient clinical studies to compare the effectiveness of natural products compared to approved therapeutics for the treatment of CVDs. Further long-term studies are needed to accelerate the potential of using natural products for these diseases. Despite this undoubted beneficence on CVDs, there are no strong breakthroughs supporting the implementation of natural products in clinical practice. Nevertheless, they are promising agents in the supplementation and co-therapy of CVDs.

## 1. Introduction

Cardiovascular diseases (CVDs) are a group of diseases of the heart and blood vessels that contribute most to morbidity and mortality in the human population [1,2]. Atherosclerosis and arterial thrombosis lead to ischemic damage of different organs such as the heart, brain, kidneys, and eyes, which can induce different failures of these organs [3]. The incidence of CVDs doubled in the last three decades, from 271 million in 1990 to 523 million in 2020, with an extremely high mortality rate of over 32% [4]. Over the past 30 years, mortality from CVDs has steadily increased. Today, one person dies every half a minute from CVDs indicating the devastating fact that one-third of all deaths in the world are due to CVDs [5]. Among the main modifiable risk factors that contribute to the development and prognosis of CVDs are a combination of different psychosocial factors: socioeconomic, behavioral, unhealthy diet, physical inactivity, illicit substance use, smoking, and environmental risk factors are of most importance.

Other nonmodifiable factors may also affect the risk of CVDs, such as genetic predisposition, ethnicity, gender, and age [6].

The clinical presentation of CVDs can vary from asymptomatic in patients with atherosclerosis [7], or often with arterial hypertension [8,9], or manifest as unspecified symptoms such as weakness, light-headedness, and nausea, or classic symptoms such as chest pain in patients with coronary artery disease (CAD) [10] or acute coronary syndromes (i.e., acute myocardial infarction) [11,12]. Different etiologic and clinical symptoms of CVDs share some common features at the cellular and molecular levels: chronic inflammation [13], mitochondrial dysfunction [14,15,16], and oxidative damage [17] to biomolecules including proteins, lipids, and nucleic acids. These factors are believed to be a progressive process that may occur as early as childhood [18].

Numerous studies in the past decades have been performed to develop better therapeutic strategies, but current medications for CVDs mainly target disease symptoms like therapeutics for CAD disease [19,20,21,22], acute myocardial infarction [23], atrial fibrillation [24,25,26], chronic heart failure [27,28], and arterial hypertension [29,30,31,32], respectively. Physicians should be careful in choosing the right kind of treatment depending on the type of disease that a patient has. Especially since certain therapeutics are not effective enough in the treatment of certain CVDs or show intolerance or side effects. Therefore, it is important to improve prevention and early diagnosis and develop therapeutic options to reduce the currently very high risk of CVDs. In recent years, the search for active ingredients from natural products and plant sources for the treatment, prevention and/or supportive therapy of various types of cardiovascular disease has become a hotspot.

The World Health Organization (WHO) estimates that approximately 75% of the world medical market consists of phytomedicine [2]. Numerous therapeutics approved by the Food and Drug Administration (FDA) used today to treat the most common CVDs have been extensively studied in preclinical and clinical studies. The efficacy of herbal medicine has been carefully reviewed in the preclinical field; no comparative studies have been found to confirm the efficacy of natural products compared to FDA-approved therapeutics for the treatment of CVDs.

Thus, this review’s goal is to highlight the most investigated natural products in the therapy of common CVDs, alongside conventional clinical therapies.

## 2. Materials and Methods

### Search Methodology

Data for this comprehensive review were obtained from electronic databases such as MedLine, PubMed, Web of Science, Scopus, and Google Scholar. The following MeSH terms were used for the search: “Cardiovascular diseases/prevention and control”, “Cardiovascular disease/treatment/natural products”, “Natural products/isolation and purification”, “Coronary heart disease/therapy/natural products”, “Myocardial infarction, treatment, natural products”, “Phytotherapy/methods”, “Phytotherapy/adverse effects”, “Action potentials/drug effects”, “Atrial fibrillation” “Valvular heart disease”, “Antihypertensive agents/pharmacology”, “Heart failure/drug therapy”, “Atherosclerosis/treatment/natural products”, “Ischemic heart disease/drug effects”, and “Vascular dysfunction and disease”. Only papers written in English that included the potential mechanisms of natural bioactive compounds in some common cardiovascular diseases were selected. Duplicate papers, communications, and studies that included homoeopathic preparations were excluded.

## 3. Most Frequent Cardiovascular Diseases

**Figure 1:**
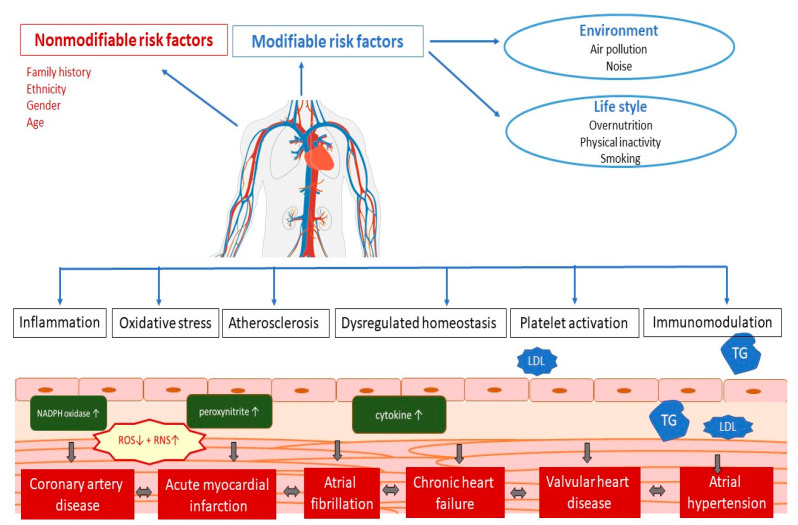
Pathophysiology of cardiovascular disease. LDL—low-density cholesterol; TG—triglycerides; ROS—reactive oxygen species; RNS—reactive nitrogen species.

CVDs is an umbrella term for all diseases of the heart and circulation [1]. The pathophysiology of the occurrence of CVDs depends on a whole range of different factors (Figure 1). Numerous studies have shown that several potential mechanisms, including endothelial dysfunction, inflammation, oxidative stress, atherosclerosis, dysregulated hemostasis, cardiac stress, and epigenetics, play a role in the development of vascular and cardiac damage [33]. The most common types of heart diseases are CAD including acute coronary syndromes, atrial fibrillation, chronic heart failure, valvular heart disease, arterial hypertension, and congenital heart disease [34]. Congenital heart disease, which is mostly genetically determined, includes a whole range of relatively rare heart diseases, so they will not be covered in this article.

### 3.1. Coronary Artery Disease

Coronary artery disease (CAD) is the most common CVD. Coronary atherosclerosis is a slow process that leads to the gradual intima thickening of the coronary arteries and subsequent development of atherosclerotic plaques that might be stable or prone to rupture due to inflammation. Atherosclerosis is the main factor that affects artery blood flow and leads to myocardial ischemia [7]. Coronary stenosis or occlusion may occur as a result of the formation of an intraluminal coronary thrombus [35]. Worldwide, an estimated 200 million people have CAD, and one in six deaths are caused by this disease [36]. In people with suspected CAD, the first option in a diagnosis is clinical diagnosis along with laboratory tests, electrocardiogram, exercise stress test, echocardiogram, and cardiac CT angiography [37].

#### 3.1.1. Treatment of Coronary Artery Disease Using Approved Drugs

**Figure 2:**
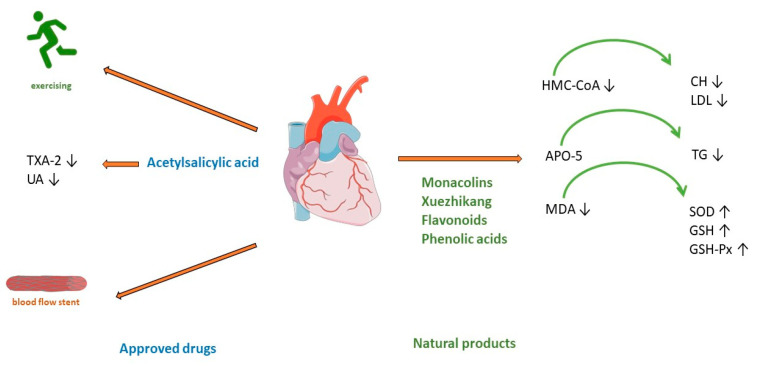
Treatment of coronary artery disease. TXA-2—Thromboxane A2; UA—uric acid; HMG-CoA—(3-hydroxy-3-methylglutaryl-coenzyme A) reductase; APO5—apolipoprotein A5; MDA—malondialdehyde; CH—cholesterol; LDL—low-density cholesterol; TG—triglyceride; SOD—superoxide dismutase; GSH—glutathione; GSH—glutathione peroxidase.

Clinical guidelines for CAD treatments recommend a combination of lifestyle changes, pharmacological treatment, and, in some cases, cardiac interventions [21,38,39]. Lifestyle modification includes a healthy diet, smoking cessation, optimal physical activity, and stress management (Figure 2). As the development of CAD includes several risk factors such as hyperlipidemia, obesity, diabetes mellitus, arterial hypertension, and smoking [16], pharmacological treatment includes target antiplatelet agents such as acetylsalicylic acid, clopidogrel, and blockers of adrenergic β receptors (beta blockers), hypolipemic drugs such as statins, fibrates or proprotein convertase subtilisin/kexin type 9 (PCSK-9) inhibitors, calcium channel blockers, organic nitrates, and various antihypertensive drugs (Figure 2) [21].

Drugs inhibit cyclooxygenase, an enzyme necessary to produce prostaglandins. Acetylsalicylic acid inhibits one of the cyclo-oxygenase enzymes, which catalyzes Thromboxane A2 (TXA-2) protein synthesis and consequently completely abolishes the formation of TXA-2 protein and reduces platelet aggregation [22]. In the acute phase, 150–320 mg of acetylsalicylic acid per day is recommended; for long-term use, the values are between 75 and 150 mg per day [20]. Acetylsalicylic acid is contraindicated in patients with an increased risk of bleeding or gastric ulcers [20].

Today, there are precise indications for invasive treatment of CAD such as percutaneous coronary interventions, meaning the implantation of a small tube called a stent into the artery (Figure 2). Stents are designed to prevent arteries from re-occlusion [40,41]. In some cases, improving coronary blood flow can be bypassed using part of the internal thoracic arteries [40]. Over time, CAD can also lead to heart failure and arrhythmias [42].

#### 3.1.2. Treatment of Coronary Heart Disease Using Natural Products

Red yeast rice has been used as a herbal supplement for lowering cholesterol and lipoprotein in human blood. It is made by fermenting white rice with the yeast *Monascus purpureus*. For many years, it has been used for flavoring, coloring, and preserving food in traditional Chinese medicine [43]. One of the more important components of this extract is monacolin K (Table 1). Monacolin K is chemically like the cholesterol-lowering drug lovastatin. It acts by competitively inhibiting HMG-CoA (3-hydroxy-3-methylglutaryl-coenzyme A) reductase, the rate-limiting enzyme of the pathway of cholesterol synthesis (Figure 2). A meta-analysis of 6663 patients (from 20 randomized clinical trials) treated with red yeast rice extract showed a reduction in low-density cholesterol (LDL) [44]. The applied dose varies from 4.8 to 24 mg of monacolin K (1200–2400 mg of red yeast rice). The advantage of this treatment shows a significant reduction in the incidence of kidney injury and liver abnormalities compared with standard statin therapy [44]. However, research stated the limitation that reporting of adverse events was insufficient in most of studies. Thus, red yeast rice may be an effective treatment for reducing cardiovascular risk in statin-tolerant patients only when a mild profile of adverse reaction is confirmed [45]. Another meta-analysis of 15 high-quality randomized clinical trials with red yeast rice applied in doses of 200–4800 mg daily showed its efficacy and safety in the treatment of hyperlipidemia.

Hypertriglyceridemia represents an independent risk of coronary heart disease [46], but in most patients with this disease, high-intensity statin therapy is not useful because of the high incidence of statin intolerance [47], so treatment with Xuezhikang, may be a better alternative (Figure 2). Xuezhikang, an extract of *Monascus purpureus*, contains monacolins, PUFAs, flavonoids, and ergosterol. Xuezhikang is a supplementary product approved by the US Food and Drug Administration and has an excellent lowering performance on triglyceride and LDL-C levels (Figure 2). In coronary heart disease patients, 6 weeks of treatment with Xuezhikang extract (1200 mg/daily) resulted in a significant reduction in cholesterol, LDL-C, and triglycerides levels [48]. A review of 22 clinical randomized trials (most of them published in Chinese) showed that Xuezhikang is safe and effective in reducing cardiovascular events in coronary heart disease complicated by dyslipidemia [49]. In rat models of high-fructose-diet-induced hypertriglyceridemia, Xuezhikang (XZK) was compared with simvastatin. Xuezhikang had a similar effect to simvastatin in lowering LDL-C, but a significantly higher hypotriglyceridemic performance was attributed to the upregulation of apolipoprotein A5 (apoA5) via the peroxisome proliferator-activated receptor α (PPARα) signaling pathway [50]. Xuezhikang contributes to greater triglyceride reduction than simvastatin in hypertriglyceridemia rats by apoA5 elevation in hepatocytes [50]. Apo A5 is a target gene of PPARa and an important regulator of triglyceride metabolism [51].

Numerous studies have demonstrated the antioxidant effects of flavonoids. In a rat model of hyperlipidemia, the administration of flavonoids from the seed of *Amygdalus mongolica* significantly lowered total cholesterol (TC), LDL-C, and the atherosclerosis index (Figure 2) [52]. The hypocholesterolemic activity of the extract could be attributed to the fact it reduced malondialdehyde (MDA) and significantly increased activities of the antioxidant enzymes superoxide dismutase (SOD), glutathione (GSH), and glutathione peroxidase (GSH-Px) (Figure 2) [52]. In a meta-analysis of 39 prospective cohort studies (23,664 individuals with CHD), the intake of quercetin and kaempferol was linearly associated with a lower risk of CHD [53]. The lowest risk was observed in individuals whose intake was up to 12–14 mg/day of quercetin.

Four phenolic acids are major compounds present in the methanolic extract of *Quercus acutissima* fruit (QF): caffeic acid, ellagic acid, gallic acid, and protocatechuic acid [54]. A recent investigation confirmed the important role of QF in cellular functions, such as gene regulation, cytoskeleton dynamics, receptor signaling, and cellular metabolism [55]. The anti-obesity, anti-hyperlipidemic, anti-cholesterol, and anti-oxidative effects of QF are associated with the inhibition of acetylation, an important factor included in metabolic regulation (Figure 2) [56].

Saponin shows antiatherosclerosis activity by regulating lipid metabolism. A randomized controlled trial with *Panax notoginseng* saponins on 84 patients with CAD showed anti-lipidemic and anti-inflammatory effects. After 30 days of treatment with this saponin, high-density lipoprotein significantly increased, and white blood cell count decreased significantly [57]. An important mechanism of *Panax notoginseng* in vitro activity changes the methylation of miR-194, its promoter, and MAPK, FAS, RAS, and FOS, and significantly decreases the apoptosis rate of HUVECs cells [57]. The compound of *Panax notoginseng* saponin is available on drug markets as an over-the-counter drug in China and around the world [58].

Hydroxysaf flower yellow A is a c-glycosyl compound, a member of phenols, extracted from safflower (*Carthamus tinctorius* L.) which shows excellent therapeutic effects on CVDs by different mechanisms, is antioxidative, and has free radical scavenging abilities and anti-inflammatory activity. In models of atherosclerosis, it can suppress foam cell formation, vascular endothelial cell dysfunction, vascular smooth muscle cell proliferation and migration, and platelet activation by regulation of the reverse cholesterol process, fatty acids synthesis, and regulation of oxidative stress parameters [59]. Hydroxysaf flower yellow A reduces vascular inflammation by regulating the expression of NF-kappaB, Bax/Bcl-2, and TLR4/Rac1/Akt, PI3K/Akt/mTOR signaling pathways [59].

Polyphenol, quercetin obtained from different natural sources, is a potent anti-atherosclerotic compound which inhibits oxidized LDL by activating sirtuin 1 (SIRT1) and reducing NOX2 and NOX4 [60]. The results also indicated that quercetin regulated endothelial NO synthase and reduced reactive oxygen species formation [60]. Numerous biological mechanisms of quercetin have been discovered; for example, it attenuates the expression of p47phox and NADPH-related oxidative damage in the aortas of high-fat-diet-fed apolipoprotein E-deficient mice (Table 1) [61].

Polyhydroxynaphthoquinone echinochrome A, a natural pigment of marine origin, is known for its anti-inflammatory, antibacterial, and antioxidant effects. In a clinical study of 140 patients with atherosclerosis, a low dose of echinochrome normalized lipid metabolism, restored antioxidant status, reduced atherosclerotic inflammation, and decreased epithelial dysfunction [62]. Echinochrome protects human cells from the negative effects of the radical by the scavenging superoxide anion, mimicking the reaction of superoxidase (Table 1) [62]. Echinochrome a is heretofore a commercially available compound that has been applied to medical usage and approved by the Ministry of Health of the Russian Federation [63].

**Table 1:** The most representative bioactive compounds and their major effects in the treatment of coronary heart disease.

Component	Source	Chemical Structure Depiction (Molecular Formula) ^1^	Biological Activity	Reference
Monacolin K	*Monascus purpureus*	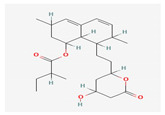 (C_24_H_36_O_5_)	inhibit HMG-CoA, lower LDL	[44]
Xuezhikang		-	lower cholesterol, LDL, TG PPARa patway	[48,50,51]
Flavonoid	*Amygdalus mongolica*	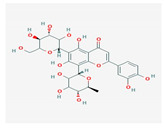 (C_27_H_30_O_15_)	lower cholesterol, LDL reduce MDA; increase antioxidant enzymes	[52]
Phenolic acid	*Quercus acutissima*	-	anti-obesity, anti-hyperlipidemic; anti-cholesterol anti-oxidative	[56]
Saponin	*Panax notoginseng*	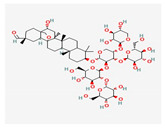 (C_58_H_97_O_27_)	changes the methylation of miR-194; anti-lipidemic anti-inflammtory	[57]
Hydroxysafflower yellow A	*Carthamus tinctorius*	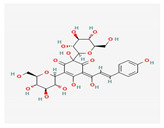 (C_27_H_32_O_16_)	regulate expression NF-kappaB, Bax/Bcl-2; anti-inflammatory, anti-oxidative	[59]
Quercetin	*Fruits*	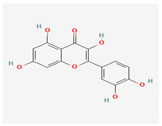 (C_15_H_10_O_7_)	activate SIRT1, reduce NOX2/NOX4	[60,61]
Echinochrome A	*Scaphechinus mirabilis,* *Spatangus purpureus*	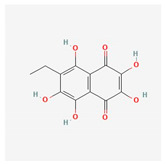 (C_12_H_10_O_7_)	normalizes lipid metabolism; restores antioxidant status; reduces atherosclerotic inflammation; decreases epithelial dysfunction	[60,61]

^1^ Chemical structure depiction (molecular formula) is taken from PubChem, an open chemistry database at the National Institutes of Health (NIH).

### 3.2. Acute Myocardial Infarction

Acute myocardial infarction occurs when the blood supply to the heart is interrupted. In this situation, the heart is no longer supplied with sufficient oxygen and nutrients, so the muscle begins to die. In many cases, myocardial infarction is not fatal, especially if patients receive early treatment [11]. Myocardial infarction is the leading cause of death worldwide, with a prevalence approaching 3 million people [12].

#### 3.2.1. Treatment of Acute Myocardial Infarction Using Approved Drugs

**Figure 3:**
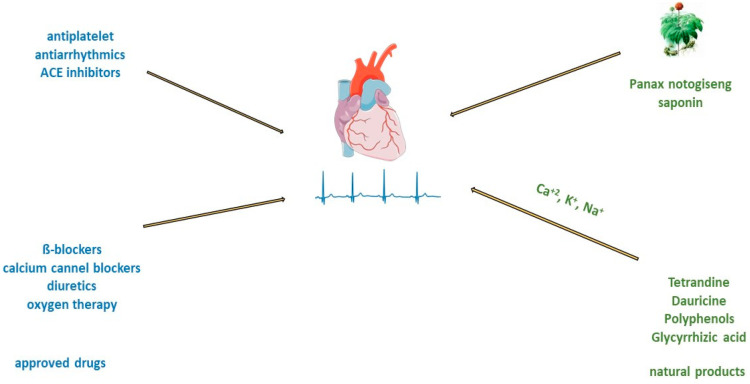
Treatment of acute myocardial infarction and atrial fibrillation with approved drugs or natural products.

The type of acute myocardial infarction (AMI) depends on the degree of coronary artery occlusion (Figure 3). The traditional recommendation for patients is to take one nitroglycerin dose sublingually, 5 min apart, for up to three doses before admission to the emergency department [64]. After AMI, it is crucial to improve cardiac function and prevent postinfarction pathophysiologic remodeling [11]. Timely revascularization of the heart after AMI depends on the infarct size; therefore, an adequate reaction of physicians is very important. Standard treatment includes the use of antiplatelets and/or anticoagulants, beta-blockers, antiarrhythmics, opiate analgesics, antihypertensives such as angiotensin-converting enzyme (ACE) inhibitors, diuretics or calcium channel blockers, and oxygen therapy. Even prognosis most often depends on the type of AMI and administration of thrombolytic treatment or PCI [23]. Consequently, many patients in which this approach is used still progress to cardiac hypertrophy and heart failure.

#### 3.2.2. Treatment of Acute Myocardial Infarction Using Natural Products

Saponin from *Panax notoginseng* exerts a cardioprotective effect in acute myocardial infarction [65]. In traditional medicine, the freeze-dried extract of Panax notoginseng for intravenous administration is used in the clinic for the prevention and treatment of cerebral ischemic injuries [66]. In addition, preclinical studies have shown the antioxidant and anti-inflammatory properties of this saponin [67,68]. Administration of Panax notoginseng injection to patients with myocardial infarction improved survival and cardiac function and decreased infarct size by direct inhibition of platelet aggregation and improved endothelial cell migration and angiogenesis (Figure 3). Panax notoginseng treatment significantly lowers lactate dehydrogenase and cardiac troponin I concentrations in the plasma of mice with MI. The mechanism of Panax notoginseng is manifested through the phosphorylation of AMPK and CaMKII in cardiomyocytes which induces autophagy [65].

Salvianolic acid B extracted from Salvia miltiorrhiza Bunge, promote angiogenesis in the marginal zone of MI by increasing the expression of VEGF [69]. In large myocardial infarction of rats, pretreatment with salvianolic acid B promotes the differentiation of mesenchymal stem cells into endothelial cells and has greater effects than the angiotensin-converting enzyme inhibitor benazepril [70].

In hyperlipidemic animals with myocardial ischemia/reperfusion, hydroxysafflower yellow A inhibited the NF-κB signaling pathway, TLR4 signaling pathway, and phosphorylation of p38 [71]. Experimental acute myocardial ischemic models reduced serum levels of inflammatory factors such as TNF-alpha, IL-1β, and IL-18, reduced NLRP3 inflammasome expression, and induced autophagy [72]. Hydroxysafflower yellow A improved antioxidant capacity and decreased apoptosis, and mitigated myocardial ischemia/reperfusion injury by inhibiting the activation of the JAK2/STAT1 pathway in adult male Sprague-Dawley rats (Table 2) [73].

In vitro studies have confirmed the cardioprotective properties of echinochrome expressed through antioxidant and anti-inflammatory activity [74]. In mouse hearts after MI, echinochrome treatment inhibits oxidative stress and reactive sulfur species production. Echinochrome a has been shown to suppress the catabolism of reactive sulfur species to H_2_S/HS^−^ in the left ventricle and suppress systolic dysfunction and structural remodeling [75]. Echinochrome could be a potential therapeutic for cardiac protection and/or regeneration in endothelial-mesenchymal transition-induced myocardial infarction after treatment has reduced the myofibroblast proportion and fibrosis area (Table 2) [76].

### 3.3. Atrial Fibrillation

Atrial fibrillation is a disorder of myocardial electrical conductivity that causes arrhythmia with various heart rhythms and rates [24]. As a result, too little blood is transported into the heart chambers (ventricles). This increases the risk of lung congestion and atrial thrombosis, as well as systemic thrombosis that causes a stroke. Uncontrolled atrial fibrillation can lead to chronic and acute heart failure [77]. The prevalence of atrial fibrillation ranged from 0.5% to 9% for people aged 50 to 90 years, respectively [42,78]. Causes of atrial fibrillation include sinus node dysfunction, coronary artery disease, rheumatic heart disease, arterial hypertension, hyperthyroidism, and alcohol [42]. Pathophysiological changes in atrial fibrillation include electrical remodeling, impaired atrial structure, autonomic nerve dysfunction, metabolic abnormalities, oxidative stress, etc. [78].

#### 3.3.1. Treatment of Atrial Fibrillation Using Approved Drugs

Treatment of atrial fibrillation usually includes rate and rhythm control, anticoagulation, and left atrial appendage closure. There is consensus that in patients with acute atrial fibrillation, parenteral anticoagulants such as heparin must be administered before cardioversion to reduce the risk of embolism [79]. Guidelines from various professional societies (The European Association of Cardio-Thoracic Surgery, American Heart Association (AHA)/American College of Cardiology (ACC)/Heart Rhythm Society (HRS)) recommend catheter ablation to restore sinus rhythm in patients with atrial fibrillation [80]. Patients with atrial fibrillation have an impaired quality of life and an increased risk of stroke, heart failure, cardiomyopathy, and acute coronary syndrome [79].

Administration of beta-blockers and calcium channel blockers is recommended as a first-line treatment for rate control of atrial fibrillation (Figure 3) [24]. Oral or intravenous application of different antiarrhythmic drugs, amiodarone, digoxin, flecainide, and ibutilide, increase the likelihood of reversion to sinus rhythm and can cause ventricular arrhythmias [25,26,81,82]. In addition, there are limitations such as that flecainide and propafenone should not be used in people with ischemic heart disease [25,26]. Treatment with verapamil, diltiazem, and digoxin may control heart rate, but they are unlikely to restore sinus rhythm [82]. On the other side, the long-time application of amiodarone can cause hepatotoxicity, interstitial lung disease, and thyroid dysfunction [83]. Therefore, the search for antiarrhythmic drugs from natural sources has been one of the priorities of scientists in recent years.

#### 3.3.2. Treatment of Atrial Fibrillation and Natural Compounds

There are many electrolytes in the human body; however, some of them, such as potassium, calcium, and sodium, play an important role in regulating signal transduction and ion transport across cell membranes. In patients with atrial fibrillation, due to electrolyte imbalance, the expression of ion channel proteins as well as gene transcription is altered, and fibrosis develops [84].

In an animal model of middle cerebral artery occlusion, saponin extracted from the roots of *Panax notoginseng* has significant antiarrhythmic and antiplatelet effects, regulates glycoprotein Ib-α, and reduces von Willebrand factor (VWF)-mediated platelet adhesion [85]. Myocardial tissue from the right and left atria of patients with atrial fibrillation after treatment with saponin increases in mitochondrial respiration rate [86]. The other group of saponins, ginsenosides, exert antiarrhythmic effects by modulating intracellular Ca^2+^ signaling through the inhibition of Ca^2+^ channels [87], or by regulating sodium, potassium, and calcium channels [88], or inhibiting collagen deposition in cardiomyocyte (Figure 3) [89].

Alkaloids are widely distributed in advanced plants and contain at least one nitrogen group. One of them, berberine, inhibits the occurrence of atrioventricular reentrant tachycardia by regulating potassium and calcium ion channels and cyclic nucleotide-gated cation channels activated by hyperpolarization [90], or prolongs action potential duration and the effective refractory period in cardiac myocytes of rabbits [91]. Another alkaloid, tetrandrine, is antiarrhythmic by the inhibition of calcium, potassium, and sodium channels. An in vitro study of tetrandrine at a dosage of 100 µmol/L in rat cardiomyocytes, reduced Ca^2+^ influx into the sarcolemma and inhibited Ca^2+^ uptake into the sarcoplasmic reticulum by inhibiting ATP [92]. The significantly low dosage of tetrandrine, 15 µmol/L, increased the opening frequency and prolonged the opening time of calcium-activated potassium channels [93]. In a concentration-dependent manner (25, 125, 250, 400, 1000, and 2500 μmol/L) guanfacine blocked the L-type calcium channel and inhibited potassium currents in rat ventricular myocytes [94]. Dauricine reduced intracellular Ca^2+^ concentration by Na^+^-K^+^-ATPase and Ca^2+^-Mg^2+^-ATPase activation [95]. Matrine at a high concentration of 100 μM inhibited the expression of the human ether-a-go-go-related gene (hERG), encoded the rapidly activating, delayed rectifier potassium channel (IKr) important for cardiac repolarization, and at a low concentration of 1 μM, martine promoted hERG expression in rat cardiomyocytes. Indeed, matrine prolonged the action potential duration and the effective refractory period of cardiomyocytes [96].

Polyphenols are secondary metabolites widely distributed in the skin, roots, and leaves of fruits and medicinal plants. In vitro, cardiac arrhythmias caused by oxidative stress and calcium overload were significantly reduced in guinea pigs’ ventricular myocytes after treatment with resveratrol. Resveratrol reduced oxygen-free radical production, prevented the activation of calmodulin-activated protein kinase II, and inhibited L-type calcium channels [97]. Hydrogen-peroxide-induced ischemic arrhythmias in ventricular myocytes were reduced after resveratrol treatment by decreasing sodium concentration and reversing the sodium–calcium exchange current [98]. Puerarin protected rats’ ventricular myocytes against ischemia and reperfusion injury by regulating the calcium-activated potassium channel and activating protein kinase C [99].

Glycyrrhizic acid can inhibit sodium influx of cardiac myocytes during depolarization, slow down conduction velocity, raise the rate of the action potential, and reduce the amplitude of action potential (Table 3) [100].

### 3.4. Chronic Heart Failure

Heart failure is a chronic, long-term condition in which the heart can no longer provide sufficient minute volume. This leads to circulus viciousness in terms of fluid retention starting from the legs, abdomen, and lungs to general edema (anasarca) in association with other symptoms of chronic heart failure. Chronic heart failure has increased to an estimated 37.7 million people, and almost 50% of these patients die within 5 years after diagnosis [101]. The risk increases with age, obesity, diabetes, smoking, alcohol abuse, or cocaine use. The guidelines of the American College of Cardiology Foundation (ACCF)/American Heart Association (AHA) defined chronic heart failure based on ejection fraction as preserved, intermediate, and heart failure reduced ejection fraction [101]. Additionally, in pathogenesis, myocardial interstitial fibrosis contributes to left ventricular dysfunction defined by the diffuse, disproportionate accumulation of collagen in the myocardial interstitium and activation of multiple molecular signaling pathways, such as endothelial dysfunction, hypertrophy of cardiomyocytes, and cardiac inflammation [10].

#### 3.4.1. Treatment of Chronic Heart Failure Using Approved Drugs

**Figure 4:**
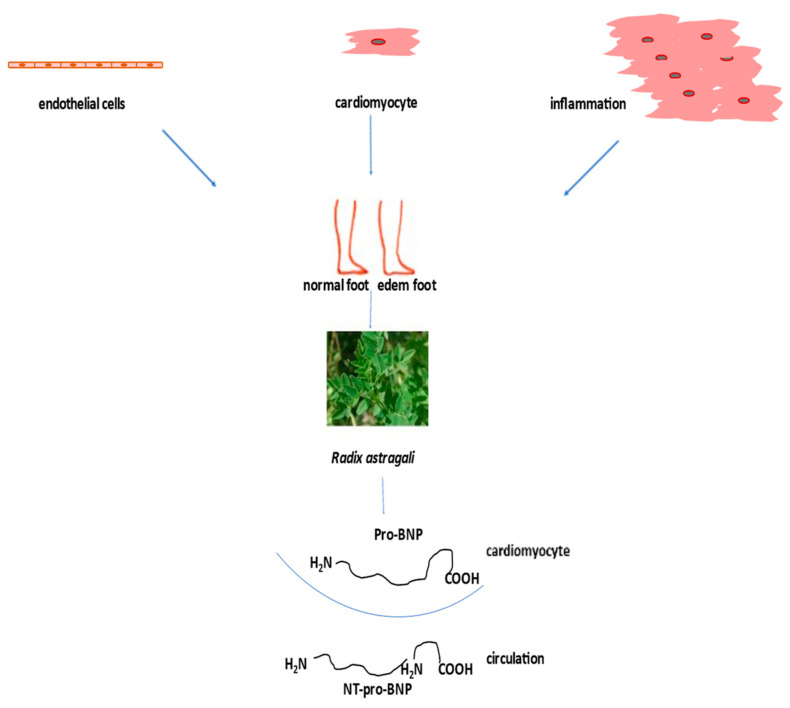
Treatment of heart failure with naturally derived astragaloside IV.

According to the guidelines for the diagnosis and treatment of acute and chronic heart failure, the following pharmacotherapeutic groups are recommended: drugs for the modulation of the renin-angiotensin-aldosterone (RAAS) and sympathetic nervous systems with ACE inhibitors or an angiotensin receptor-neprilysin inhibitor (ARNI), beta-blockers, mineralocorticoid receptor antagonists (MRA), loop and thiazide diuretics, and newly introduced gliflozins (inhibitors of sodium-glucose transport proteins 2) and ivabradine [101]. The side effects of high-dose diuretics can lead to low blood pressure, electrolyte disorders, and worsening of heart failure symptoms. Aldosterone antagonists can induce hyperkaliemia (Figure 4).

#### 3.4.2. Treatment of Chronic Heart Failure and Natural Compounds

Heart failure is usually associated with different risk factors such as chronic inflammation, hypertension, type 2 diabetes mellitus (T2DM), obesity, coronary artery disease, and sarcopenia. Since the incidence of heart failure has increased in recent decades, and there are no adequate pharmacological therapies, there is an urgent need to test nonpharmacological strategies, such as the use of natural products, to improve clinical outcomes in these patients.

One of the most commonly used natural products to treat heart failure when other medications do not help is digoxin, a secondary glycoside. Its side effects include digestive problems, confusion, and visual disturbances [102].

Saponin astragaloside IV (AS-IV) is one of the main active ingredients of the aqueous extract *Radix Astragali Huangqi* (The Root of Astragalus membranaceus var. mongholicus) injected into heart failure patients. This injection improves cardiac function by increasing left ventricular ejection fraction and decreasing stroke volume [103]. A meta-analysis of seven randomized clinical trial with 550 patients in total has shown that Di’ao Xinxuekang capsules, steroidal saponins, extracted from *Dioscorea panthaica*, have a better protective effect on heart failure patients than isosorbide dinitrate [104]. Also, the incidence of adverse events was lower in Di’ao Xinxuekang capsule-treated patients. In a clinical trial, a total of 512 patients with chronic heart failure were divided into a control/placebo group and a treated group. The treated group took capsules of qili qiangxin and saponin for 12 weeks. Treatment significantly decreased plasma N-terminal pro-B-type natriuretic peptide, and improved left ventricular ejection fraction (Figure 4) and quality of life [105]. In chronic heart failure patients, Shenmai injection, ginsenoside saponin, improved left ventricular diastolic function [106].

Fuzi (Radix Aconiti Praeparata) is an important ingredient in many traditional Chinese medicine recipes and belongs to the group of alkaloids. Studies on the therapeutic potential of aconitine have been conducted for more than a decade in heart failure models. In vitro studies showed a significant cardiotonic effect on cells with heart failure, as well as a marked improvement in hemodynamic parameters in rats with acute heart failure [107]. The results of this experiment showed that the +dp/dtmax, LVEF, and LVFS of rats with heart failure were significantly increased after intravenous injection of aconitine, but that this sometimes triggered ventricular extrasystoles. Recent studies show hepatotoxic and neurotoxic properties of aconitine [108,109].

Cardiac oxidative stress is increased during heart failure. There is increased expression of nicotinamide adenine dinucleotide phosphate (NADPH) oxidases (NOX), which increases the production of reactive oxygen species (ROS) [63]. Increased ROS activates several kinases, p38MAPK, ERK, and c-Jun N-terminal kinase, which can induce cellular apoptosis [110]. Increased production of ROS increases the production of several cytokines, transforming growth factor (TGF)-β, interleukin (IL)-6, IL-1β and tumor necrosis factor (TNF)-α, pro-inflammatory factors that cause fibrosis in myocytes [111].

Numerous polyphenols, flavonoids, flavanols, anthocyanins, and flavanones exhibit cardioprotective properties of heart failure [112]. The cytoprotective role of catechin, the anthocyanidins cyanidin, delphinidin, and quercetin in ischemia cardiomyocytes improves cell viability [113].

Cyanidin-3-galactoside, cyanidin-3-arabinoside, and cyanidin-3-glucoside independently decrease cell apoptosis in H9c2 myoblasts [114]. Hypaconitine and glycyrrhetinic acid modulate the metabolic pathway in rats suffering from chronic heart failure, increase the expression of vascular endothelial growth factor A and fibroblast growth factor 2, decrease lipid levels, and attenuate the expression of eNOS protein [115].

Polyphenols likely play a direct role in modulating heart failure by reducing oxidative stress and inflammation in the heart [112].

Therefore, evidence suggested that a variety of polyphenols could act in synergistic or additive ways in different underlying signaling pathways of heart failure, as synthesized in Table 4.

### 3.5. Valvular Heart Disease

The heart’s primary function is to pump blood throughout the body. During a typical human lifespan, the heart valves open and close more than 3 billion times. The valves control the unidirectional flow of blood through the heart. Heart valve disease occurs when one or more heart valves no longer open or close properly. More than 40 million people worldwide live with heart valve disease [117].

#### 3.5.1. Treatment of Valvular Heart Disease Using Approved Drugs

Treatment of valvular heart disease depends on the symptoms, but it always considers clinical guidelines including antiplatelet agents and statins to reduce thrombosis and prevent atherosclerosis [116]. If an irregular heart rhythm, atrial fibrillation, is present, antiarrhythmic therapy is sometimes needed as well [22].

In many cases, heart valve surgery may be needed to repair or replace a diseased heart valve. More than 180,000 heart valve replacement surgeries are performed each year. Heart valve surgery is usually performed through transcatheter valve repair, which is an alternative to traditional open valve replacement surgery. This procedure can be performed with a balloon-expandable or self-expanding valve [117].

#### 3.5.2. Treatment of Valvular Heart Disease Using Natural Products

Heart valve diseases involve inflammatory reactions and increase oxidative stress [118]. The three layers fibrosa, spongiosa, and ventricular are the main components of valve function, diastole, and systole extension. Therefore, preservation of the structures of these layers plays an important role in the function of valves.

Fucoxanthin treatment has been shown to effectively protect heart valve interstitial cells by the inhibition of Akt/ERK-related signaling pathway, reducing valve calcification and apoptosis and restoring cell viability [119]. In vivo experiments in dogs treated with fucoxanthin also showed significant recovery of their echocardiographic parameters after 6 to 24 months [119]. Fucoxanthin is a carotenoid with high anti-oxidative, anti-inflammatory, anti-cancer, and anti-hyperuricemia effects [119].

### 3.6. Arterial Hypertension

Arterial hypertension is a condition in which the pressure of peripheral middle and small arteries against the walls is permanently elevated [9]. The incidence of patients with arterial hypertension ranges from about 35% in general to higher rates in patients who have side effects or do not respond to traditional therapy [120]. The number of adults with hypertension increased from 594 million in 1975 to 1.13 billion in 2018 [8]. Arterial hypertension can develop in any segment of life, and it often occurs as a result of genetic predisposition, stress, some other diseases, and an unhealthy lifestyle.

#### 3.6.1. Treatment of Arterial Hypertension Using Approved Drugs

**Figure 5:**
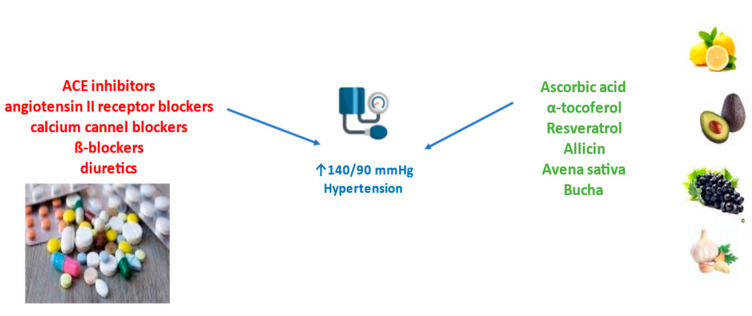
Treatment of arterial hypertension with approved drugs or natural products.

The various classes of antihypertensive drugs include thiazide diuretics, beta-blockers, ACE inhibitors, angiotensin II-receptor blockers, calcium channel blockers, alpha-blockers, and a combination of some of these drugs (Figure 5), according to the guidelines for the prevention, detection, evaluation, and management of arterial hypertension in adults [121].

Diuretics help the body to eliminate excess salt (sodium) and water. Long-term use of diuretics increases the risk of developing gout. Another side effect of diuretic therapy can be the depletion of mineral potassium [29,31].

Beta-blockers, also known as beta-adrenergic blockers, block the action of the hormone epinephrine (adrenaline) on β1 and β2 receptors leading to decreased myocardial contractility and frequency as well as decreasing renin release in renal juxtaglomerular cells, thus lowering blood pressure. Possible side effects of beta blockers include bradycardia or atrioventricular blocks, heart block, insomnia, sleep disturbances, constipation, and sexual and/or erectile dysfunction [30].

Angiotensin-converting enzyme (ACE) inhibitors reduce the effects of the hormone angiotensin II. They block the conversion of angiotensin I to angiotensin II with the inhibition of angiotensin convertase. Angiotensin II has a strong constricting effect on blood vessels in many ways. This hormone also stimulates salt and water retention in the body, which can increase blood pressure. Possible side effects of this therapeutic agent may include dizziness, irritable dry cough, hyperkalemia, and in rare cases, acute kidney failure [29].

Calcium channel blockers reduce the amount of calcium that enters the cells of the heart and blood vessel walls. Calcium enters these cells through special pores called ion channels. When these channels are blocked, the amount of calcium entering decreases, the blood vessels relax, and the heart receives more oxygenated blood. The most common side effects are swelling, redness of the skin, palpitations, constipation, and a slowing of heartbeat rate [31].

Epinephrine and norepinephrine activate alpha-1 adrenergic receptors in vascular smooth muscle, causing vasoconstriction of blood vessels. Vasoconstriction is the main cause of increased systemic arterial blood pressure and peripheral resistance. Therapy with alpha-adrenergic antagonists such as prazosin blocks alpha receptors and causes vasodilation, which lowers blood pressure [32]. Adverse effects of nonselective alpha-blockers include hypotension, weakness, tachycardia, and tremulousness [32]. Severe arterial hypotension can lead to heart ischemia as well as ischemic damage to major organs.

#### 3.6.2. Treatment of Arterial Hypertension and Natural Products

Excess production of ROS, reactive nitrogen species (RNS), or failure of antioxidant defenses causes endothelial damage, vascular dysfunction, cardiovascular remodeling, kidney dysfunction, excitation of the sympathetic nervous system, activation of immune cells, and systemic inflammation. All these changes play an important role in the pathophysiology of hypertension. A large amount of data in the literature published in the past supposes the use of antioxidants as therapeutic agents to treat arterial hypertension. Antioxidants can modify vascular function and influence the redox outcomes implicated in the pathology processes of hypertension. Several experimental studies focus on the development of drug candidates that could reduce blood pressure.

Various vitamins have significant antioxidative properties (Figure 5). Ascorbic acid reduces intrarenal oxidative stress, increases ACE, and endothelial function, increases eNOS activity, and decreases levels of ROS and RNS sources that improve vascular function and lower blood pressure [122,123]. Vitamin E, α-tocopherol in a dose-dependent manner, reduces blood pressure by regulating the mitochondria generation of superoxide anion and hydrogen peroxide production [124]. In a patient with essential hypertension, vitamin D supplementation significantly reduces systolic and mean blood pressure [125]. In this open-label clinical study, 173 patients with essential hypertension participated, and vitamin D was administered in doses of 50,000 IU/week, and 1000 IU/day in patients with vitamin D levels < 30 ng/mL for 8 weeks.

Polyphenolic compounds like resveratrol in hypertensive conditions significantly reduce regional and systemic blood pressure by improving the bioavailability of nitric oxide [126], preserving endothelium [127], regulating antioxidative enzyme activity, reducing inflammation and apoptosis, and ameliorating morphological changes in the aorta [126], heart, and kidney [128,129]. Also, resveratrol is the most studied polyphenol in clinical trials. In a systematic review and meta-analysis of 17 randomized, controlled clinical trials on the impact of resveratrol on blood pressure, it was concluded that, as an active compound, resveratrol was only effective in high daily doses (≥300 mg/day) and in diabetic patients [130]. The most important obstacle of resveratrol is low bioavailability after oral intake. Thus, different types of carries for RSV have been developed, including liposomal particles [131]. Additionally, resveratrol precursors, like polydatin and pterostilbene [132], have been investigated as agents that are more promising. Over eighteen years, a cohort study of 11,056 participants conformed that the intake of foods rich with polyphenols lowered hypertension risk [133].

Saponin and ginsenoside decrease hypertension with the inhibition of vascular remodeling of small artery ends and can stimulate endothelial-dependent vessel dilatation [134,135]. In the dysfunctional human pulmonary artery, endothelial cells’ astragalus attenuated hypoxia-induced proliferation and apoptosis and regulated inflammatory cytokines’ production and expression of proteins p27, p21, Bax, caspase-9, and caspase-3 [136].

Supplemented with quercetin, flavonoids, based on meta-analysis (587 patients in total were included, and supplementation was in doses 100–1000 mg/daily), showed a statistically significant effect on lowering blood pressure in doses higher than 500 mg/daily [137]. Quercetin improves endothelial function due to a NO-dependent mechanism, decreases levels of ET-1, and also produces vasodilation by endothelium-independent pathways [138].

Allicin, a thioester of sulfenic acid, is the primary active compound of *Allium sativum* and possesses important hypotensive properties. It has been reported that increased production of nitric oxide results in smooth muscle relaxation and vasodilation [139]. The effects of garlic have been observed in hypertensive patients for 12 weeks. In those patients, treatment with garlic pearls significantly reduced 8-hydroxi-2-deoxigenase, levels of nitric oxide and lipid peroxidation, and increased levels of antioxidative vitamins [140].

Aristolochic acid, aristoloside, magnoforine, oleanolic acid, hederagenin, and tannins are the components of the plant *Aristolochia manshuriensis* used as a diuretic for the treatment of oedema in hypertensive patients [141].

*Avena sativa* has high essential unsaturated fatty acid content, soluble dietary fiber, particularly beta-glucan, and high concentration antioxidants, which have been shown to lower blood cholesterol and glucose absorption, which can reduce inflammatory state, type 2 diabetes, and hypertension [142]. Extract of *Capparis decidua* also showed antihypertensive activities through endothelium-dependent and Ca^2+^ antagonist pathways [143].

*Buchu* has been used for two species, *Agathosma betulina* and *Agathosma crenulata*, containing different compounds like flavonoids, diosmin, quercetin, hesperidin, and rutin. *Buchu* consumption significantly lowers serum aldosterone levels, reduces elevated blood pressure, and inhibits the release of potent cytokines like interleukin-6 and tumor necrosis factor-α (Table 5) [144].

A total of 884 randomized controlled intervention trials involving 883,627 participants, studying 27 different types of micronutrients, showed that supplementation with 7 micronutrients lowered both systolic and diastolic blood pressure [145].

## 4. Discussion

CVDs differ in their etiopathogenesis and clinical symptoms. However, they share some common features at the cellular and molecular levels: chronic inflammation, mitochondrial dysfunction, and oxidative damage to biomolecules such as proteins, lipids, and nucleic acids. Blood vessels are involved in the regulation of vascular blood flow. Nitric oxide, one of the important vasodilating agents, can interact with ROS under conditions of increased oxidative stress characteristic of CVDs, reducing the bioavailability of NO and affecting the alteration of endothelial function. An imbalance between the production of relaxing endothelial factors and contractile endothelial factors results in endothelial dysfunction, which is a hallmark of many CVDs. While there are many chemical drugs available to treat cardiovascular disease, some of them have side effects or do not respond well enough. The therapeutic limits of approved therapy are characterized by a whole range of negative features, and often have allergic effects, so they must be excluded [19,20,21,22,23,24,25,26,27,28,29,30,31,32]. Conventional therapy of CVDs requires an individual approach to patients, while on the other hand, the application of natural products does not have that kind of limitation. Nowadays, natural products are used in many CVDs. Many synthetic drugs originated from herbal medicines. Therefore, the use of various natural products that could protect the body from increased ROS and RNS production can significantly help protect against the development of CVDs [134,146]. Sometimes, natural remedies must be used with caution because they can manifest cytotoxic, cardiotoxic, and neurotoxic effects [108,109]. Natural products’ different mechanisms slow down the progression of CVDs. They could scavenge free radicals, improve anti-oxidative defense, decrease the levels of inflammatory cytokines, improve autophagy, and inhibit apoptosis.

The results of clinical trials show that acetylsalicylic acid reduced platelet aggregation by inhibiting cyclo-oxygenase enzymes in patients with CAD [22]. Recent studies have shown that the active ingredients of natural products, monacolins, have excellent effects on cholesterol, triglyceride, and LDL-C levels by inhibiting HMG-CoA or activating PPARα [43]. A meta-analysis of 6663 patients showed that this natural product significantly reduced the incidence of kidney and liver injury [44]. This study suggests that monacolins could be more effective than conventional drugs. The therapeutic efficacy of flavonoids and phenolic acids is shown in the improvement of antioxidant capacity and effects on the regulation of lipid levels [52,56]. Studies showed that quercetin regulates endothelial NO synthase and attenuates the expression of NOX2, NOX4, and p47phox. The results suggest that quercetin reduced oxygen species formation [60,61]. The clinical study of 140 patients with atherosclerosis found echinochrome A reduced inflammation and restored antioxidant status with the scavenging superoxide anion [62]. This was confirmed in patients with CAD after chronic consumption of cranberry juice, rich in polyphenols and anthocyanins, by reduced carotid femoral pulse wave velocity [147]. These studies suggested that natural products have cardioprotective effects on patients with CAD.

Clinical studies have shown that the saponin from *Panax notoginseng* has a therapeutic effect on AMI. The results of previous studies have shown improvement in cardiac function and a reduction in infarct size with direct inhibition of platelet aggregation [65]. In preclinical studies, treatment with *Panax notoginseng* was shown to significantly decrease lactate dehydrogenase and troponin T by regulating the phosphorylation of AMPK [67,68]. Recent preclinical studies have found that hydroxysafflower yellow A has therapeutic effects on acute myocardial infarction. Hydroxysafflower yellow A was shown to reduce TNF-alfa, IL-1β, IL-18, and NLRP3, improve antioxidant capacity, and decrease apoptosis [71,72]. A large number of meta-analyses have been published regarding the efficiency of polyunsaturated omega-3 fatty acids in prevention after myocardial infarction [147].

The use of various medications is recommended for patients with acute atrial fibrillation: anticoagulants, antiarrhythmics, beta-blockers, and calcium channel blockers. With the discovery of the therapeutic effect of natural products in the treatment of acute atrial fibrillation, the demand for these herbal/animal sources has increased significantly. Different groups of saponins and alkaloids are expected to have antiarrhythmic effects by regulating sodium, potassium, and calcium channels. Concerning cardiac arrhythmias, organic acid and glycyrrhizic acid can block sodium and calcium ion channels, prolonging the duration of the action potential [100]. Natural products affect the expression and function of genes responsible for coding ion channel proteins.

The current regimen requires a direct assessment of the risk–benefit ratio using the recommended pharmacotherapeutic groups for the treatment of acute and chronic heart failure. Previous studies have shown that active ingredients of natural products could be used for therapy in future. In a clinical study of 512 patients with chronic heart failure, saponin was found to improve left ventricular ejection fraction by regulating N-terminal pro-B-type natriuretic peptide [105,106]. Numerous preclinical studies in acute and chronic heart failure found that alkaloids and polyphenols derived from medical plants work through various mechanisms to counteract oxidative stress [147].

The use of conventional therapy for the regulation of high blood pressure has expanded in recent years. This includes different classes of antihypertensive drugs: thiazide diuretics, beta-blockers, ACE inhibitors, angiotensin II-receptor blockers, calcium channel blockers, alpha-blockers, and a combination of some of these drugs [121]. Each of them has different mechanisms of action, so some regulate the elimination of water and sodium [29,31]. Others decrease renin release in renal juxtaglomerular cells by blockade beta receptors [30]. Calcium channel blockers relax blood vessels by decreasing the influx of calcium ions into the cells [30]. There is an important link between complex oxidation reactions and the development of atrial hypertension. Compounds derived from natural products work through various mechanisms to counteract oxidative stress. Vitamins reduce blood pressure by regulating the production of hydrogen peroxide and reducing the formation of superoxide anions [124]. Antihypertensive effects of polyphenols, saponin, and ginsenoside were evidenced by the preservation of endothelium, regulation production of cytokines, antioxidative enzymes, bioavailability of nitric oxide, and the expression of proteins Bax, Bcl, p27, p21, caspase-9, and caspase-3 [126,128,129,134,136]. The cardioprotective effects of foods rich in polyphenols in epidemiological studies were shown to improve endothelial function and plasma lipid profiles [147].

Numerous studies show great potential in traditional medicine. Isolation of active components of plants and their extracts, as well as studies of their mechanisms of action, may open new perspectives for the formulation of new drugs. Natural products have great advantages in the treatment of CVDs due to their safety profiles. In the last 20 years, about one-third of all FDA-approved drugs were based on natural products and their derivatives. In addition, people are more willing to use natural products to prevent/treat various diseases, so they have great potential when combined with conventional therapy. Traditional medicine focuses on treating people, not just their symptoms. Nevertheless, despite this undoubted beneficence on CVDs, there is no strong evidence for using natural products in standard clinical practice.

## 5. Conclusions

This review collates varying levels of evidence on the effects of different natural products in the prevention and treatment of most frequent CVDs. The combination of traditional therapies and natural products could lead to a synergistic effect so that the efficacy of individual drugs could be markedly improved. Despite the lack of information about the exact mechanisms of action for many natural compounds/extracts, their long-term usage in the traditional medicine of many different countries encourages their use in the treatment of various CVDs. However, due to insufficient data about the toxicity, exact doses, and possible interactions, people should be careful with their usage. Also, the effect of a single natural substance on CVDs could be small, and it would be important to take into consideration future research on a combination of natural substances. However, it should be carried out carefully as natural complexes contain multiple active compounds, and due to unknown mechanism of actions, some of them may attenuate and/or potentiate the effect of each other. The development of more effective natural drug-based cardiovascular medicine implies the application of genomics, proteomics, metabolomics, and other technologies to further understand the molecular pathogenesis of CVDs and mechanisms of action of natural products, alone and/or in combination with traditional therapies. A limitation of the current review is the inclusion in the study of evidence from preclinical/clinical pharmacological models and meta-analyzes. On the other hand, the inclusion and analysis of these in vitro, in vivo, and meta-analyzes could be a strong point of this review, as it opens new therapeutic potential of natural bioactive compounds in the therapy of CVDs. Further long-term studies are needed to corroborate the large wealth of data in the literature available on the role of natural products in the treatment of different types of CVDs to determine the implications for clinical applications. Despite the methodological limitations related to narrative reviews, it is possible to infer that no strong breakthroughs support the implementation of natural products in clinical practice, but they are promising agents in the supplementation and co-therapy of CVDs.

## Figures and Tables

**Table 2:** The most representative bioactive compounds and their major effects in the treatment of myocardial infarction.

Component	Source	Chemical Structure Depiction (Molecular Formula) ^1^	Biological Activity	Reference
Ginsenoside Rb1	*Panax notoginseng*	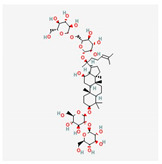 (C_54_H_92_O_23_)	decreased infarct size by direct inhibition of platelet aggregation and improved endothelial cell migration and angiogenesis. lower lactate dehydrogenase and troponin I; induces autophagy through phosphorylation of AMPK and CaMKII in cardiomyocytes	[65]
Ginsenoside Rd		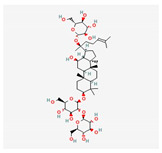 (C_48_H_82_O_18_)		[65]
Ginsenoside Rg1		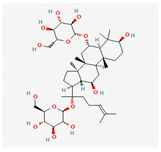 (C_42_H_72_O_14_)		[65]
Salvianolic acid B	*Salvia miltiorrhiza*	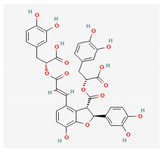 (C_36_H_30_O_16_)	exchanging expression VEGF; differentiation of mesenchymal stem cells into endothelial cells	[70]
Hydroxysafflower yellow A	*Carthamus tinctorius* L.	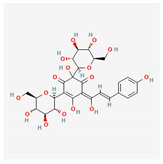 (C_27_H_32_O_16_)	inhibition of phosphorylation p38, NF-κB, and TLR4 signaling pathway; reduction TNF-α, IL-1β, IL-18; Inhibition JAK2/STAT1 pathway	[71,72,73]
Echinochrome A	*Scaphechinus mirabilis, Spatangus purpureus*	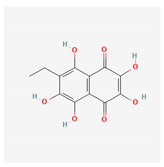 (C_12_H_10_O_7_)	suppress the catabolism of reactive sulfur species to H_2_S/HS^−^; cardiac protection and/or regeneration	[60,61]

^1^ Chemical structure depiction (molecular formula) is taken from PubChem an open chemistry database at the National Institutes of Health (NIH).

**Table 3:** The most representative bioactive compounds and their major effects in the treatment of atrial fibrillation.

Components	Source	Chemical Structure Depiction (Molecular Formula) ^1^	Biological Activity	References
Saponin	*Panax notoginseng*	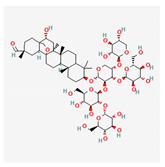 (C_58_H_97_O_27_)	antiarrhythmic, antiplatelet, regulates glycoprotein Ib-α, reduces platelet adhesion	[85]
			increases mitochondrial respiration rate	[86]
			Regulate sodium, potassium, and calcium channels; inhibit collagen deposition in cardiomyocyte	[87,88,89]
Berberine	*European* *barberry*	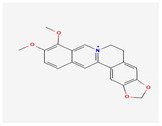 (C_20_H_18_NO_4_^+^)	regulate potassium and calcium ion channels	[90,91]
Tetrandrine	*Stephania tetrandra*	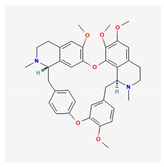 (C_38_H_42_N_2_O_6_)	inhibit calcium, potassium, and sodium channels	[92,93]
Resveratrol	Red grapes	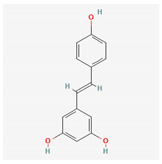 (C_14_H_12_O_3_)	activation of calmodulin-activated protein kinase II, and inhibition of L-type calcium channels	[97,98]
Glycyrrhizic acid	*Glycyrrhiza glabra*	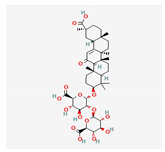 (C_42_H_62_O_16_)	reduce action potential myocytes	[100]

^1^ Chemical structure depiction (molecular formula) is taken from PubChem, an open chemistry database at the National Institutes of Health (NIH).

**Table 4:** The most representative bioactive compounds and their major effects in the treatment of heart failure.

Components	Source	Chemical Structure Depiction (Molecular Formula) ^1^	Biological Activity	References
Astragaloside IV	*Astragali Huangqi* *Astragalus membranaceus*	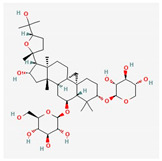 (C_41_H_68_O_14_)	increasing left ventricular ejection fraction and decreasing stroke volume	[103,104,106]
*Fuzi*	*Aconiti praeparata*	-	improvement hemodynamic parameters	[108,109]
Flavonoid	*Amygdalus mongolica,*	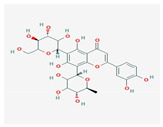 (C_27_H_30_O_15_)	reduce cytokines	[112]
Catechin	Fruits	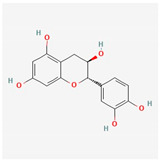 (C_15_H_14_O_6_)	improves cardiomyocytes viability	[113]
Glycyrrhizic acid	*Glycyrrhiza glabra*	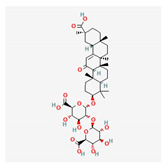 (C_42_H_62_O_16_)	increase the expression of vascular endothelial growth factor A and fibroblast growth factor 2	[116]

^1^ Chemical structure depiction (molecular formula) is taken from PubChem, an open chemistry database at the National Institutes of Health (NIH).

**Table 5:** The most representative bioactive compounds and their major effects in the treatment of hypertension.

Components	Source	Chemical Structure Depiction (Molecular Formula) ^1^	Biological Activity	References
Ascorbic acid	fruits	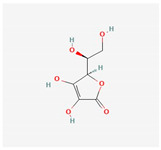 (C_6_H_8_O_6_)	increases eNOS activity and decreases the amounts of ROS and RNS	[122,123]
α-tocopherol	papaya peppers	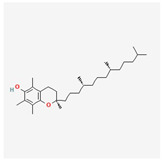 (C_29_H_50_O_2_)	superoxide anion and hydrogen peroxide production	[124]
Resveratrol	red grapes	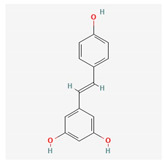 (C_14_H_12_O_3_)	anti-oxidative anti-inflammatory preserved endothelium improved bioavailability of nitric oxide	[126,127,128,129]
Quercetin	fruits vegetables	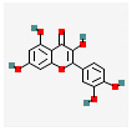 (C_15_H_10_O_7_)	improves endothelial function	[137,138]
Ginsenosides	*Panax notoginseng*	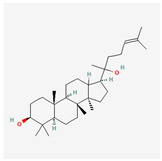 (C_30_H_52_O_2_)	stimulate endothelial-dependent vessel dilatation	[134,135]
Allicin	*Allium sativum*	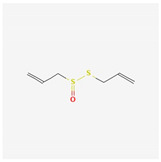 (C_6_H_10_OS_2_)	increased production of nitric oxide; relaxation/vasodilation of smooth muscle	[139]
	*Avena sativa*	-	lover cholesterol	[142]
	*Capparis decidua*	-	Ca^+2^ antagonist pathways	[143]
Buchu	*Agathosma betulina* *Agathosma crenulata*	-	lower serum aldosterone levels	[144]

^1^ Chemical structure depiction (molecular formula) is taken from PubChem, an open chemistry database at the National Institutes of Health (NIH).

## Data Availability

Data is contained within the article.
